# Machine Learning‐Enhanced Nanoparticle Design for Precision Cancer Drug Delivery

**DOI:** 10.1002/advs.202503138

**Published:** 2025-06-19

**Authors:** Qingquan Wang, Yujian Liu, Chenchen Li, Bin Xu, Shidang Xu, Bin Liu

**Affiliations:** ^1^ School of Biomedical Sciences and Engineering Guangzhou International Campus South China University of Technology Guangzhou 511442 P. R. China; ^2^ Department of Chemical and Biomolecular Engineering National University of Singapore 4 Engineering Drive 4 Singapore 117585 Singapore

**Keywords:** biomaterial, drug delivery, machine learning, nanomedicines

## Abstract

In recent years, nanomedicine has emerged as a promising approach to deliver therapeutic agents directly to tumors. However, despite its potential, cancer nanomedicine encounters significant challenges. The synthesis of nanomedicines involves numerous parameters, and the complexity of nano–bio interactions in vivo presents further difficulties. Therefore, innovative approaches are needed to optimize nanoparticle (NP) design and functionality, enhancing their delivery efficiency and therapeutic outcomes. Recent advancements in Machine Learning (ML) and computational methods have shown great promise for precision cancer drug delivery. This review summarizes the potential use of ML across all stages of NP drug delivery systems, along with a discussion of ongoing challenges and future directions. The authors first examine the synthesis and formulation of NPs, highlighting how ML can accelerate the process by searching for optimal synthesis parameters. Next, they delve into nano–bio interactions in drug delivery, including NP–protein interactions, blood circulation, NP extravasation into the tumor microenvironment (TME), tumor penetration and distribution, as well as cellular internalization. Through this comprehensive overview, the authors aim to highlight the transformative potential of ML in overcoming current challenges, assisting nanoscientists in the rational design of NPs, and advancing precision cancer nanomedicine.

## Graphical abstract

Machine Learning (ML) is revolutionizing cancer nanomedicine by optimizing nanoparticle (NP) design and drug delivery. This review summarizes ML applications across all stages of NP drug delivery, along with a discussion of ongoing challenges and future directions. By tackling key challenges in drug delivery, ML‐driven approaches enhance precision and efficiency, paving the way for advances in nanomedicine.

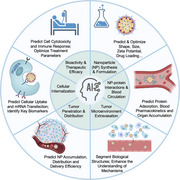

## 1 Introduction

Cancer treatment remains one of the most formidable challenges in medicine, driving a continuous search for more effective and targeted treatments.^[^ ^1^, ^2^, ^3^, ^4^ ^]^ In recent years, nanomedicine has emerged as a promising approach in this quest, leveraging the unique properties of nanomaterials to revolutionize cancer therapy.^[^ ^5^, ^6^, ^7^, ^8^, ^9^, ^10^, ^11^ ^]^ By delivering therapeutic agents directly to tumor sites, nanomedicine aims to enhance the efficacy of treatments while minimizing side effects.^[^ ^10^, ^12^, ^13^ ^]^ The ability to fine‐tune the physicochemical characteristics of nanoparticles (NPs), such as their material composition, size, shape, surface chemistry, and targeting ligands, has enabled the creation of highly specialized delivery vehicles tailored for cancer treatment. However, despite its potential, the field of cancer nanomedicine faces significant challenges.^[^ ^14^, ^15^, ^16^, ^17^, ^18^, ^19^, ^20^, ^21^ ^]^ The synthesis and formulation of new nanomedicines involves numerous parameters that must be carefully balanced to create effective therapies.^[^ ^22^, ^23^, ^24^, ^25^ ^]^ Additionally, the complexity of nano‐bio interactions presents further challenges.^[^ ^26^, ^27^, ^28^, ^29^, ^30^, ^31^, ^32^ ^]^ The heterogeneous nature of tumors further complicates the behavior and efficacy of NPs in vivo due to the dynamic interactions within the tumor microenvironment (TME).^[^ ^10^, ^33^, ^34^, ^35^ ^]^ These challenges highlight the need for innovative approaches to optimize NP design and functionality, ensuring that nanomedicines achieve their full potential in targeting and treating cancer.

To address these challenges, recent advancements^[^ ^36^, ^37^, ^38^, ^39^, ^40^, ^41^, ^42^ ^]^ in Machine Learning (ML) and computational methods have shown great promise in advancing cancer nanomedicine.^[^ ^43^, ^44^, ^45^, ^46^, ^47^, ^48^ ^]^ ML, a subset of Artificial Intelligence (AI), enables computational systems to learn patterns from data and make predictions or decisions without being explicitly programmed.^[^ ^37^, ^49^ ^]^ ML approaches can be broadly categorized into supervised learning, unsupervised learning, semi‐supervised learning, and reinforcement learning, depending on the type of data and the learning objective.^[^ ^36^, ^50^ ^]^ Among these, supervised learning is the most commonly applied in nanomedicine, where labeled datasets are used to train models that predict specific outcomes (**Table** **1**). For example, tree‐based models, which operate by learning hierarchical decision rules from data, are particularly popular due to their interpretability, robustness, and strong performance across diverse tasks. They have been widely used to predict the physicochemical properties of NPs,^[^ ^44^, ^51^ ^]^ nano–bio interactions,^[^ ^52^, ^53^, ^54^ ^]^ and clinical therapeutic outcomes.^[^ ^55^ ^]^ Another foundational and widely adopted ML approach is the neural network, which plays a central role in modern ML. Inspired by biological neurons in the human brain, neural networks consist of layers of interconnected units that can model complex, nonlinear relationships. They serve as the backbone of many state‐of‐the‐art ML architectures and are especially powerful in analyzing high‐dimensional, heterogeneous data.^[^ ^49^ ^]^ In nanomedicine, neural networks have been extensively applied to support the optimization of NP design and the modeling of complex nano–bio interactions.^[^ ^56^, ^57^, ^58^, ^59^ ^]^ These advanced tools can handle the numerous variables involved in NP synthesis and formulation, modeling the intricate relationships between them to optimize design and functionality.^[^ ^24^, ^43^, ^44^, ^59^, ^60^, ^61^, ^62^, ^63^, ^64^, ^65^, ^66^ ^]^ Furthermore, ML and computational methods can not only predict nano‐bio interactions but also extract critical knowledge to enhance our understanding of the heterogeneous nature of tumors and elucidate the mechanisms behind dynamic interactions within the TME.^[^ ^47^, ^54^, ^58^, ^67^, ^68^, ^69^, ^70^, ^71^ ^]^

**Table 1:** Summary of supervised ML algorithms applied to NP design for precision cancer drug delivery.

ML Category	Model	Task	Strengths	Limitations	Applications
Linear	Linear Regression	REG^b)^	Simple and fast Interpretable	Limited to linear relationship Sensitive to multicollinearity	Predict drug release kinetics^[^ ^75^ ^]^ Predict protein corona adsorption^[^ ^97^, ^100^ ^]^ Predict blood pharmacokinetics^[^ ^110^ ^]^ Predict NP distribution in tumors^[^ ^70^ ^]^ Predict the delivery efficiency^[^ ^129^ ^]^ Predict cellular uptake^[^ ^98^, ^141^ ^]^ Predict immune response^[^ ^68^ ^]^
Linear	Logistic Regression	CLS^a)^	Efficient for binary classification Interpretable	Limited to linear decision boundaries Poor performance with complex data	Predict immune response^[^ ^68^ ^]^
Non‐linear (Tree‐based)	Decision Tree	CLS & REG	Captures non‐linear patterns Handles both numerical and categorical data Interpretable	Prone to overfitting Unstable with small data changes	Predict the in vivo therapeutic efficacy^[^ ^55^ ^]^
Non‐linear (Tree‐based)	Random Forest	CLS & REG	Reduces overfitting via ensembling Handles high‐dimensional data Robust to outliers and noise	Slower training Less interpretable	Predict drug loading efficiency^[^ ^44^ ^]^ Predict protein corona adsorption^[^ ^52^, ^99^, ^101^ ^]^ Predict blood pharmacokinetics^[^ ^109^, ^110^ ^]^ Predict the pulmonary immune responses and lung burden^[^ ^53^ ^]^ Predict cellular uptake^[^ ^52^ ^]^ Predict the delivery efficiency^[^ ^129^ ^]^ Identify key biomarkers for NP–cell interactions^[^ ^45^ ^]^
Non‐linear (Tree‐based)	XGBoost & LightGBM	CLS & REG	High predictive performance Built‐in regularization Efficient with large‐scale data	Sensitive to hyperparameters May overfit small datasets Less interpretable	Predict NP physicochemical properties^[^ ^51^ ^]^ Predict blood pharmacokinetics^[^ ^110^ ^]^ Predicting NP accumulation^[^ ^54^ ^]^ Predict mRNA transfection^[^ ^47^ ^]^ Predict NP cytotoxicity^[^ ^156^ ^]^ Predict immune response^[^ ^68^ ^]^ Predict the in vivo therapeutic efficacy^[^ ^55^ ^]^
Non‐linear (Distance‐based)	K‐Nearest Neighbors	CLS & REG	Simple and intuitive Non‐parametric No training phase	Sensitive to feature scaling Poor scalability Suffer from high‐dimensional data issues	Predict NP physicochemical properties^[^ ^51^ ^]^ Predict the in vivo therapeutic efficacy^[^ ^55^ ^]^
Non‐linear (Kernel‐based)	Support Vector Machines	CLS & REG	Effective in high‐dimensional spaces Robust to overfitting Works well with both linear and non‐linear decision boundaries	Sensitive to the choice of kernel Not suitable for large datasets Hard to interpret	Predict NP physicochemical properties^[^ ^51^ ^]^ Predict blood pharmacokinetics^[^ ^110^ ^]^ Predict the pulmonary immune responses and lung burden^[^ ^53^ ^]^ Predict NP accumulation in micrometastase^[^ ^67^ ^]^ Predict the delivery efficiency^[^ ^129^ ^]^ Predict cellular uptake^[^ ^98^ ^]^
Non‐linear (Bayesian Models)	Naive Bayes	CLS	Simple and ast Robust to irrelevant features	Assumes feature independence Performs poorly with correlated features	Predict the in vivo therapeutic efficacy^[^ ^55^ ^]^
Non‐linear (Bayesian Models)	Gaussian Processes	CLS & REG	Probabilistic predictions Quantifies uncertainty Works well with small data	Computationally expensive Poor scalability	Predict drug‐loading in solid lipid NPs^[^ ^192^ ^]^
Non‐linear (Neural Networks)	Multilayer Perceptron	CLS & REG	Learns complex patterns Flexible architecture Can approximate any function	Requires large datasets Prone to overfitting without proper regularization Hard to interpret	Predict NP physicochemical properties^[^ ^51^, ^56^, ^61^, ^73^, ^74^ ^]^ Predicts the UV–Vis absorbance spectra of NP^[^ ^59^ ^]^ Predict blood pharmacokinetics^[^ ^57^, ^110^ ^]^ Predict organ accumulation^[^ ^57^ ^]^ Predict the delivery efficiency^[^ ^129^ ^]^ Predict cellular uptake^[^ ^58^ ^]^ Predict mRNA transfection^[^ ^149^ ^]^ Predict the in vivo therapeutic efficacy^[^ ^55^ ^]^
Non‐linear (Neural Networks)	Other Deep Neural Networks	CLS & REG & Generation	Capable of modeling complex, non‐linear patterns Powerful in learning from unstructured data (e.g., images, sequences, texts) Scalable to large datasets End‐to‐end learning without handcrafted features	Require large datasets and extensive tuning Prone to overfitting, especially on small datasets Hard to interpret	Predict NP physicochemical and protein adsorption^[^ ^142^ ^]^ Segment blood vessels in tumor images^[^ ^46^ ^]^ Predict the cellular uptake^[^ ^144^ ^]^ Generate intratumoral NP distribution images^[^ ^128^ ^]^

^a)^ CLS refer to classification task;

^b)^ REG refer to regression task.

**Figure 1:**
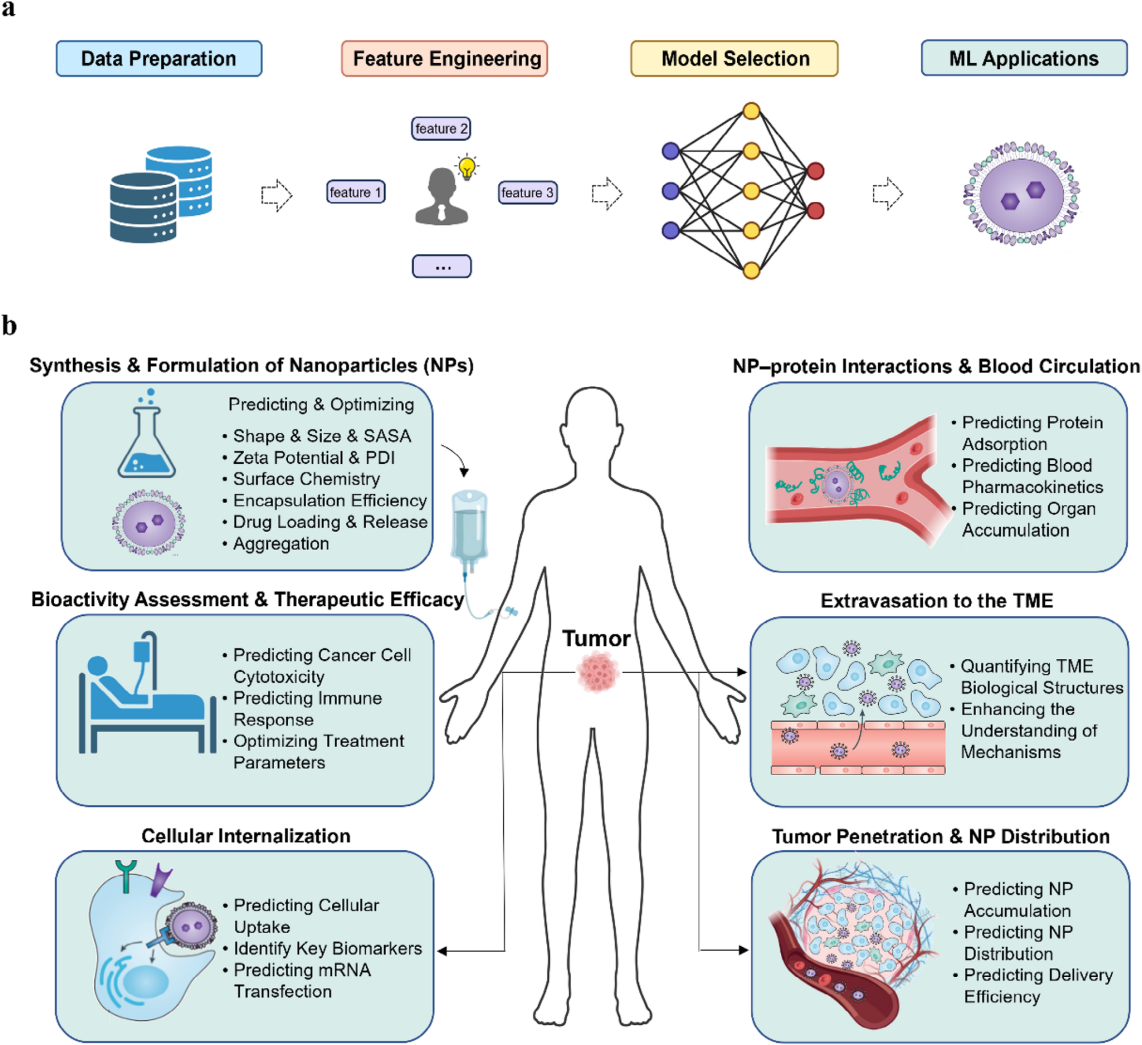
a) The workflow of applying machine learning (ML) to nanomedicine. Created with BioRender.com. b) Overview of ML‐enhanced nanomedicine drug delivery Processes. This figure illustrates the integration of ML in optimizing various stages of nanomedicine drug delivery. ML contributes significantly to the synthesis and formulation phase by predicting and optimizing parameters such as nanoparticle (NP) shape, size, solvent accessible surface area (SASA), zeta potential, polydispersity index (PDI), surface chemistry, encapsulation efficiency, and drug loading and release profiles. In the protein adsorption and blood circulation phase, ML predicts protein adsorption on NP surfaces as well as key pharmacokinetic parameters. During the extravasation to the tumor microenvironment (TME), ML quantifies tumor vascular permeability, helping us understand the mechanisms that facilitate NP transport across vascular barriers. Reproduced with permission.^[^ ^7^ ^]^ Copyright 2017, Springer Nature. In the tumor penetration phase, ML elucidates the relationship between tumor heterogeneity and the accumulation and distribution of NPs, aiming to enhance delivery efficiency. For cellular internalization, ML predicts cellular uptake, identifies key biomarkers and predicts mRNA transfection. Reproduced with permission.^[^ ^7^ ^]^ Copyright 2017, Springer Nature. Finally, ML assists in bioactivity assessment by analyzing large‐scale clinical datasets and predicting therapeutic outcomes, thereby providing a comprehensive evaluation of the efficacy and safety of nanomedicine formulations. Created with BioRender.com.

In this review, we summarize the potential use of ML in advancing nanomedicine, particularly in the design and optimization of NPs for targeted drug delivery systems. We begin by examining the synthesis and formulation of NPs, highlighting how ML can accelerate the process by searching for optimal synthesis parameters. Next, we delve into the complexity of nano–bio interactions in drug delivery, including NP–protein interactions, blood circulation, NP extravasation into the TME, tumor penetration and distribution within tumor tissues as well as cellular internalization, highlighting how ML can optimize these processes to enhance bioactivity and therapeutic efficacy (**Figure** **1**). Through this comprehensive overview, we aim to highlight the transformative potential of ML and computational methods in overcoming current challenges, assisting the rational design of NPs, and advancing the precision delivery of cancer nanomedicine.

## 2 Synthesis and Formulation of NPs

The synthesis and formulation of NPs involves precise control over numerous parameters, such as concentration, temperature, reaction time, chemical composition, etc., to achieve desired properties and functionalities. This complexity makes the traditional trial‐and‐error approach time‐consuming and resourceintensive. The introduction of ML and computational methods has revolutionized this process by providing a systematic and efficient approach to navigating the vast parameter space.

**Figure 2:**
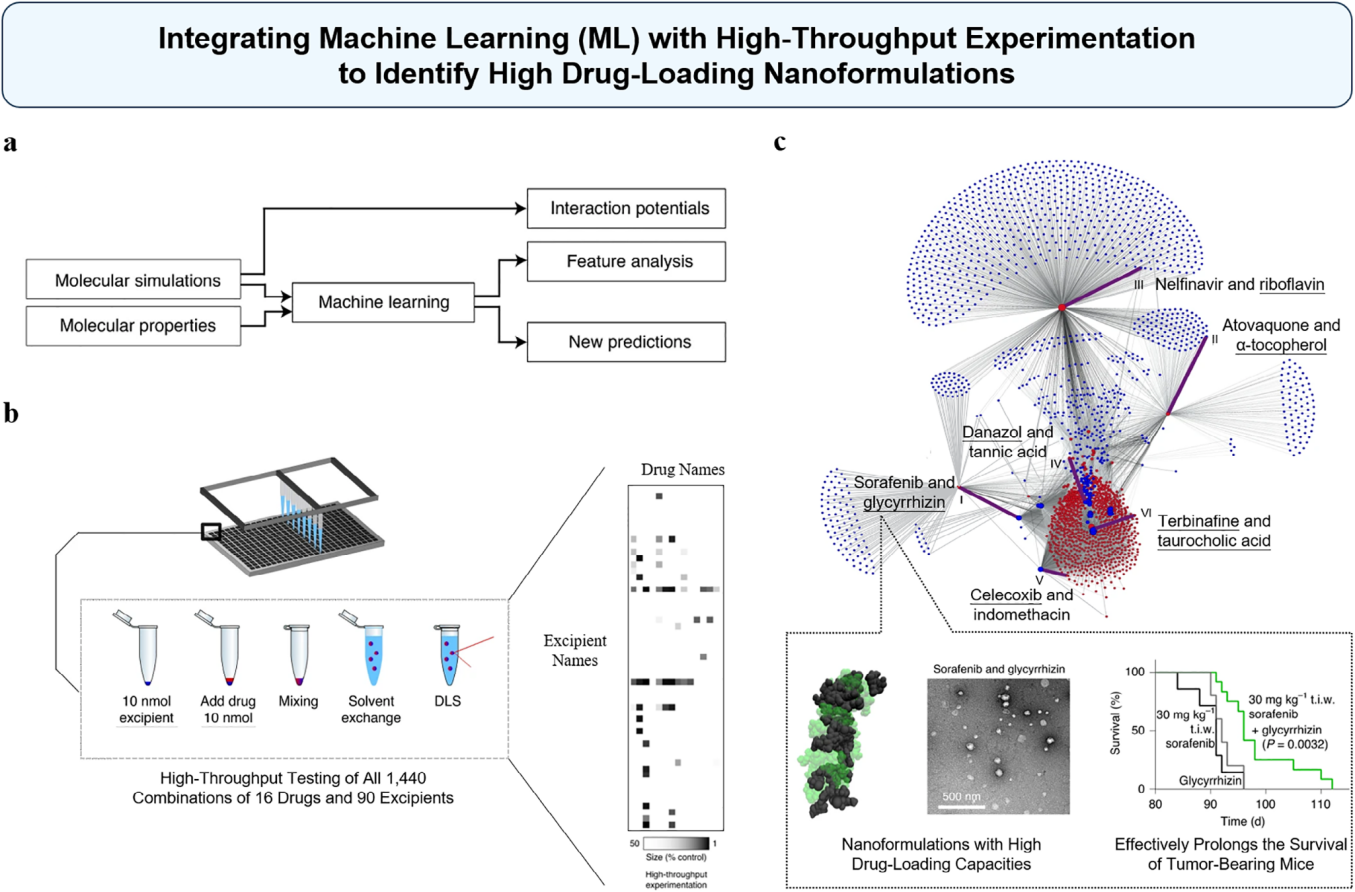
Integrating ML with high‐throughput experimentation to identify high drug‐loading nanoformulations. a) Molecular Dynamics simulation (MD) of drug–excipient systems quantified non‐covalent interaction potentials, which served as input for an ML model. This model used these interaction potentials and the molecular properties of drugs and excipients to predict drug–excipient pairs likely to form NPs. b) Left: Schematic of high‐throughput experimental workflow for creating NPs using nanoprecipitation and rapid Dynamic Light Scattering assessment. Right: High‐throughput testing of drug–excipient combinations, with a color gradient indicating NP size reduction compared to the unformulated drug. c) This figure visualizes the interaction network between drugs (red dots) and excipients (blue dots) as predicted by a computational model. The purple edges highlight specific drug–excipient formulations (I–‐VI) that were further characterized, such as Sorafenib and Glycyrrhizin (I). Node size corresponds to the number of predicted excipients or drugs that can form NPs with the respective compound. Gray lines indicate potential interactions between drugs and excipients. The bottom dashed box shows MD and Transmission Electron Microscopy (TEM) results on the left, and Kaplan–Meier analysis on the right. MD simulations map non‐covalent interactions between drugs (black van der Waals spheres) and excipients (green spheres). TEM images show NPs formed by drug–excipient co‐aggregation. Kaplan–Meier analysis shows mice treated with sorafenib–glycyrrhizin NPs have longer morbidity‐free survival compared to oral sorafenib and glycyrrhizin‐only control. Reproduced with permission.^[^ ^44^ ^]^ Copyright 2021, Springer Nature.

Through the use of ML, we can construct physicochemical property prediction models based on information such as molecular structure and properties, reaction conditions, and molecular interactions.^[^ ^24^, ^51^, ^56^, ^59^, ^62^, ^63^, ^64^, ^72^, ^73^, ^74^ ^]^ For example, ML can be combined with Molecular Dynamics simulations (MD) to predict NP encapsulation efficiency and drug loading capacity.^[^ ^43^, ^44^, ^60^, ^61^, ^75^, ^76^ ^]^ Recently, Reker et al. have integrated ML with high‐throughput experimentation to identify stable, self‐assembled drug NPs (**Figure** **2**).^[^ ^44^ ^]^ The study focused on co‐aggregating drugs with excipients, aiming to form NPs without the need for chemical synthesis. As shown in Figure 2b, first, the high‐throughput screening of drug–excipient co‐aggregation rapidly analyzed 1440 drug–excipient combinations to identify potential NP formations. Then, a Random Forest (RF) ML model was trained using the experimentally derived data points, with molecular properties and interaction potentials from MD of these drug–excipient combinations as inputs (Figure 2a). The model successfully predicted stable drug–excipient NPs with a 91% accuracy based on cross‐validation results. The predictive ML model then screened 2.1 million drug–excipient combinations, predicting 38464 (1.8%) with high potential for stable NP formation. Experimental validation identified 100 novel co‐aggregated drug NPs from these predictions. Notable examples included terbinafine–taurocholic acid NPs for antifungal applications and sorafenib–glycyrrhizin NPs for enhanced anticancer efficacy (Figure 2c). The cooperation of ML with high‐throughput experimentation significantly accelerates the development of high‐drug‐loading nanoformulations, providing an effective solution to enhance the bioavailability and therapeutic efficacy of poorly soluble drugs.^[^ ^44^ ^]^

**Figure 3:**
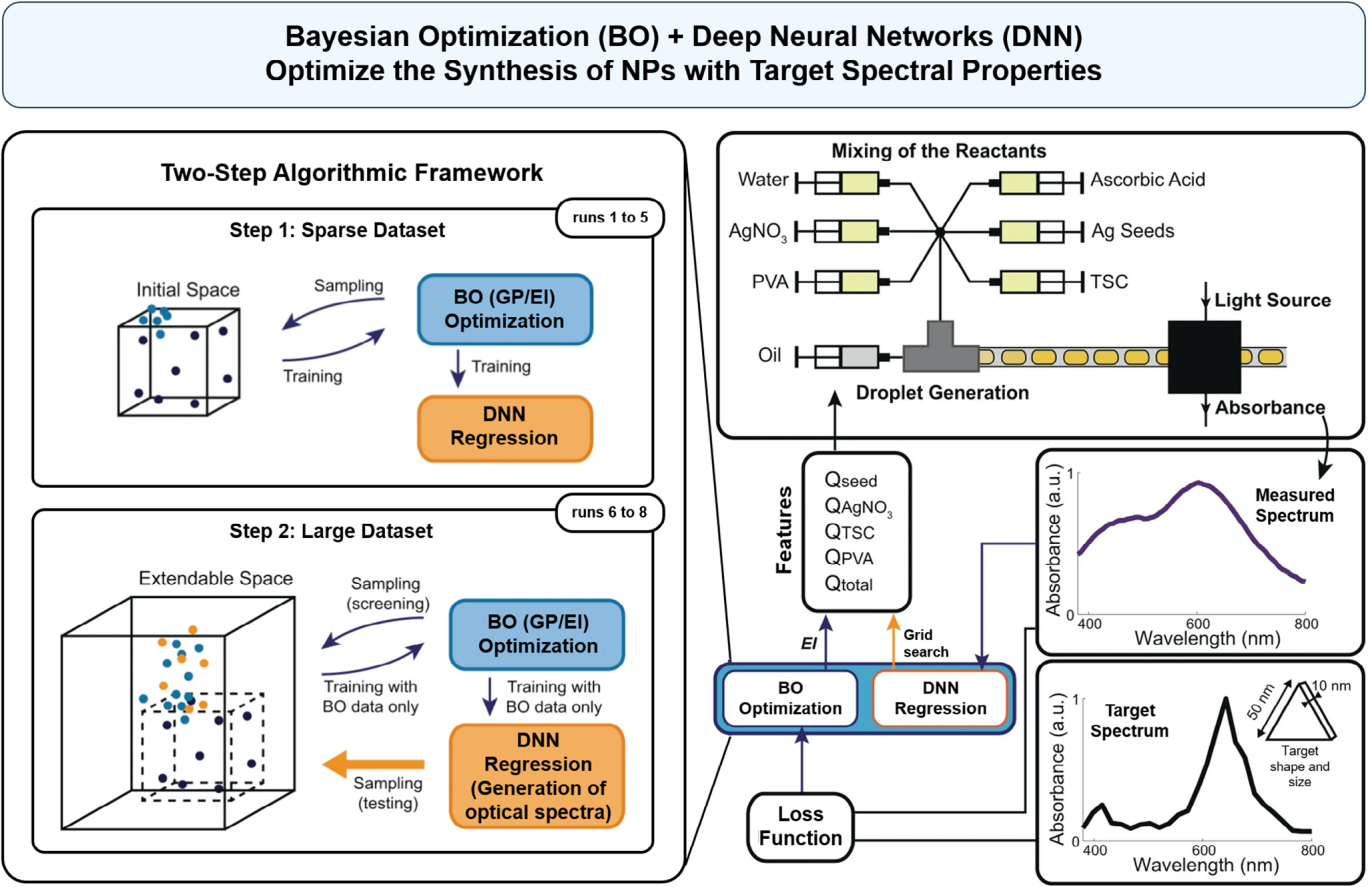
Bayesian Optimization (BO) combined with Deep Neural Networks (DNNs) optimizes the synthesis of NPs to achieve target spectral properties. Left: The two‐step optimization framework includes an initial loop (runs 1–5) where BO (blue points) samples the parameter space to train a DNN. In the subsequent loop (runs 6–8), the DNN (orange points) samples the parameter space to validate its regression function. Right: The suggested conditions from BO and DNN are tested on a droplet‐based microfluidic platform. The absorbance spectrum of each droplet is measured and compared to the target spectrum using a loss function before inputting to BO, while the fully resolved absorbance spectrum is provided to DNN. Reproduced under terms of the CC‐BY license.^[^ ^59^ ^]^ Copyright 2021, Mekki‐Berrada et al., published by Springer Nature.

We can also combine ML with optimization algorithms like Bayesian Optimization (BO) to find the optimal NP formulation with desired properties through multiple iterations. In a recent study, Mekki‐Berrada et al. have employed a novel two‐step ML framework to synthesize silver NPs with targeted absorbance spectra for imaging and tracking of biological systems (**Figure** **3**).^[^ ^59^ ^]^ The framework utilized a high‐throughput microfluidic platform combined with BO and Deep Neural Networks (DNNs) algorithms. Initially, BO was used to explore the parameter space and identify optimal conditions for the synthesis. Subsequently, the DNN, trained on data generated from the BO, predicted the conditions that minimized the loss function related to the optical properties of the NPs. After multiple iterations, the process converged to NPs with the target spectral properties. The study demonstrates the efficacy of the framework in producing NPs with desired optical properties, which are essential for enhancing imaging, tracking, and targeted release in NP drug delivery systems. Also, integrating ML with automated synthesis platforms, such as those enabled by microfluidics,^[^ ^59^, ^73^, ^77^, ^78^, ^79^ ^]^ provides a highly integrated experimental environment, allowing researchers to explore complex chemical spaces more efficiently and accurately, ultimately optimizing reaction conditions for desired properties.^[^ ^59^ ^]^ While this research optimized absorbance spectra, Liu et al. advanced NP emission properties, focusing on their behavior in the aggregated state and highlighting Aggregation‐Induced Emission (AIE) as a critical property in nanomedicine for drug delivery.^[^ ^80^ ^]^ AIE‐based NPs enhance bioimaging by allowing real‐time tracking of drug distribution and release within the body due to their strong fluorescence in aggregated states.^[^ ^81^, ^82^, ^83^, ^84^, ^85^ ^]^ This characteristic promotes the precise delivery of therapeutic agents to target cells or tissues, thereby improving treatment efficacy and reducing side effects. Their stability and brightness also make them suitable for long‐term imaging and monitoring, invaluable in advanced nanomedicine applications.^[^ ^86^, ^87^ ^]^

**Figure 4:**
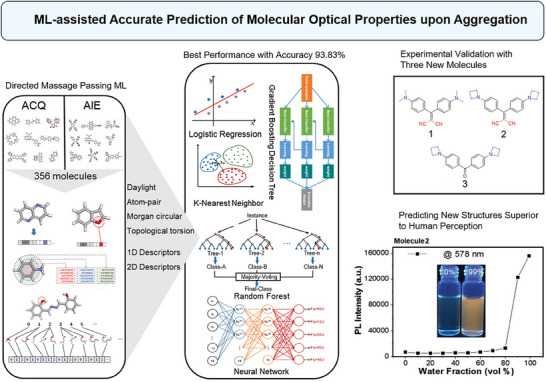
ML‐assisted accurate prediction of molecular optical properties upon aggregation. The figure illustrates the multi‐modal approach using qualitative and quantitative molecular descriptors, combined with 5 ML algorithms: Logistic Regression, K‐Nearest Neighbor, Gradient Boosting Decision Tree, Random Forest (RF), and Neural Network. The model achieved the best performance with 93.83% accuracy. The approach was experimentally validated with three new molecules, predicting new structures superior to human perception. The rightmost figure shows molecule 2 correctly predicted with Aggregation‐Induced Emission (AIE) properties, validated by photoluminescence intensity at 578 nm. Reproduced under terms of the CC‐BY license.^[^ ^80^ ^]^ Copyright 2021, Liu et al., published by Wiley‐VCH.

In a study by Liu et al., an ML framework was employed to predict the molecular optical properties of compounds in their aggregated state, focusing on both AIE and Aggregation‐Caused Quenching (ACQ) (**Figure** **4**).^[^ ^80^ ^]^ The study established a database of 356 AIE/ACQ molecules and applied various ML algorithms to predict AIE/ACQ properties. Both qualitative and quantitative molecular descriptors were used in an ensemble strategy that combined multiple prediction methods. The approach was validated by synthesizing three new molecules and comparing predictions with experimental outcomes. The multi‐modal ML approach demonstrated high predictive accuracy for AIE/ACQ properties, achieving test accuracies exceeding 90%. The synthesized molecules showed good agreement between predicted and experimental optical properties. This study highlights the potential of ML in accelerating the development of solid‐state optical materials by providing a reliable and efficient method for predicting molecular properties in aggregated states.^[^ ^80^ ^]^ In conclusion, the integration of ML and computational methods into the synthesis of NPs represents a significant advancement, accelerating the discovery process and enhancing the various properties of NPs for targeted drug delivery systems.

## 3 NP–Protein Interactions & Blood Circulation

When NPs enter a biological environment, they could be covered by a layer of biomolecules, forming what is known as a “corona”.^[^ ^88^, ^89^, ^90^, ^91^ ^]^ This corona significantly alters the size, stability, and surface properties of the NPs, which in turn determine their physiological responses. The formation of the protein corona can influence the blood circulation, biodistribution, cellular uptake, and immune response of the NPs, making it a critical factor in the development of nanomedicine.^[^ ^92^, ^93^, ^94^, ^95^, ^96^ ^]^ Understanding these interactions is essential for designing NPs that are both effective and safe for biomedical applications.

**Figure 5:**
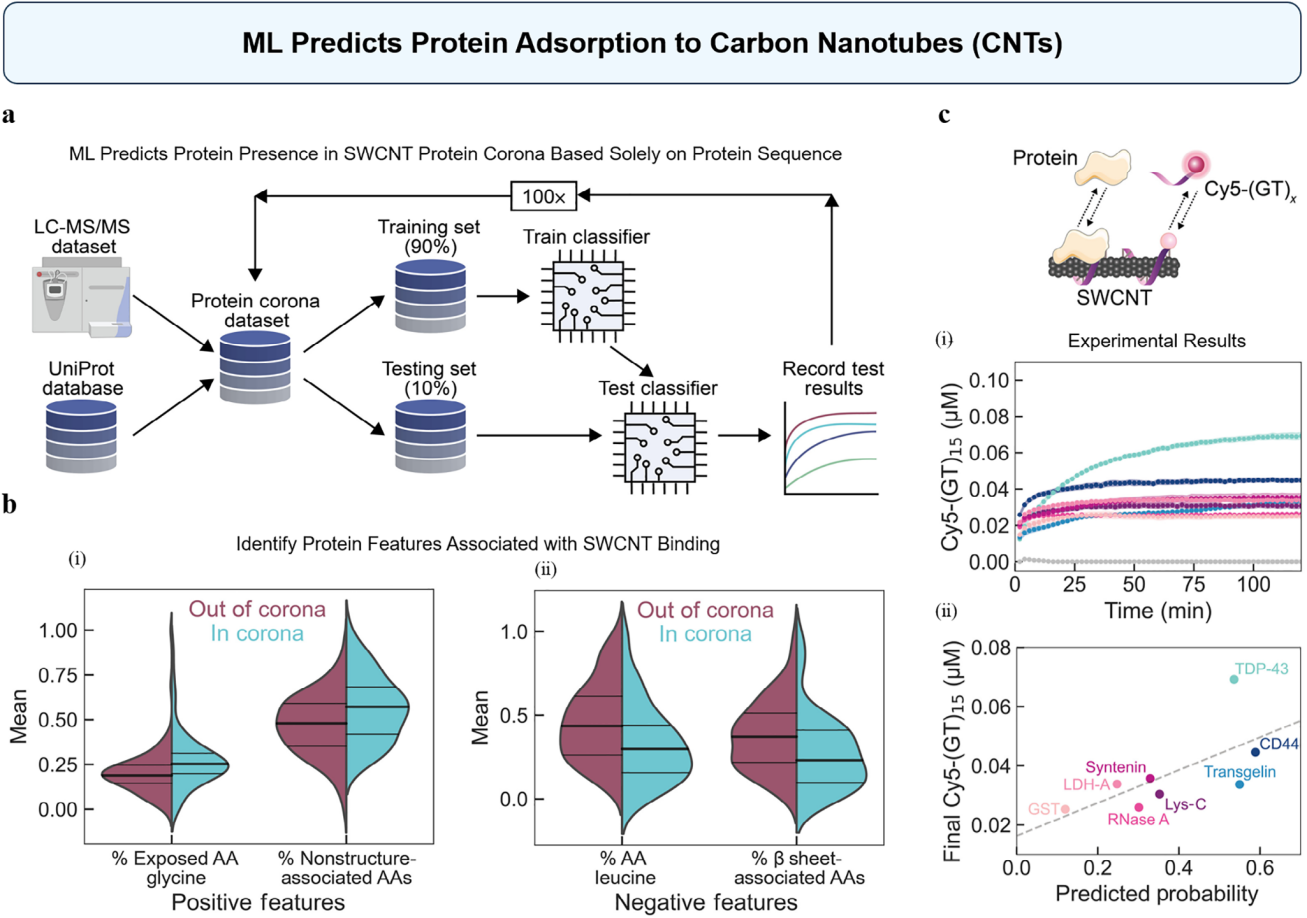
ML predicts protein adsorption to carbon nanotubes (CNTs). a) The Random Forest Classifier (RFC) workflow used a dataset combining Liquid Chromatography‐Tandem Mass spectrometry protein corona composition data with UniProt protein properties data. The dataset was split into 90% training and 10% test data. b) Positive features (i) and negative features (ii) influencing protein corona formation on (GT)15‐SWCNTs. SWCNT, single‐walled carbon nanotube. c) Protein corona dynamics for (GT)15‐SWCNTs. (i) The figure shows the concentration of desorbed Cy5‐(GT)15 ssDNA versus time by experimental results from a corona exchange assay, where ssDNA desorption serves as a proxy for protein adsorption onto the SWCNTs. (ii) Comparison of endstate‐desorbed ssDNA with the RFC‐predicted in‐corona probability for (GT)15‐SWCNTs. Proteins are predicted by the RFC to be in the corona (probability > 0.5; blue‐green colors) or out of the corona (probability < 0.5; purple‐pink colors). Reproduced under terms of the CC‐BY license.^[^ ^69^ ^]^ Copyright 2022, Ouassil et al., published by American Association for the Advancement of Science.

By utilizing ML, we can predict the types and abundance of proteins adsorbed onto NPs based on protein sequence, structure, and properties, as well as the physicochemical properties of the NPs.^[^ ^52^, ^57^, ^69^, ^97^, ^98^, ^99^, ^100^, ^101^, ^102^, ^103^, ^104^, ^105^, ^106^ ^]^ In a recent study, Ouassil et al. have developed a Random Forest Classifier (RFC) trained with mass spectrometry data to predict protein adsorption to carbon nanotubes (CNTs) based solely on the protein sequence (**Figure** **5**).^[^ ^69^ ^]^ This model achieved 78% accuracy and 70% precision, highlighting its effectiveness in predicting which proteins are likely to adsorb to CNTs. As shown in Figure 5b, the model identified key features that influence protein binding, such as a high content of solvent‐exposed glycine and non‐secondary structure‐associated amino acids, both of which increase protein binding affinity. In contrast, proteins with higher leucine content and β‐sheet structures showed lower affinity due to their rigidity. Experimental validation using a corona exchange assay confirmed the model's predictions (Figure 5c). To be specific, single‐stranded DNA (ssDNA) was labeled with a fluorescent dye (Cy5) and adsorbed onto the single‐walled carbon nanotube (SWCNT) surface, where its fluorescence was quenched. When proteins interacted with the SWCNT, they bound to the SWCNT, causing ssDNA to desorb. This desorption led to a dequenching of the Cy5 fluorescence. By tracking the fluorescence changes, the adsorption of protein to the SWCNT could be monitored. As shown in Figure 5c, the end‐state fluorescence values corresponding to protein adsorption were mostly consistent with the classifier's predictions of whether the proteins were inside or outside the corona. This computational approach not only predicts NP–protein interactions but also provides deeper insights into the fundamental mechanisms governing these interactions, which is crucial for tailoring NPs to specific biomedical applications. In silico protein corona prediction also reduces the reliance on experimental mass spectrometry‐based proteomic characterization and analysis, facilitating the development of nanotechnologies that can be implemented in biological systems.^[^ ^69^ ^]^

Blood circulation represents another critical stage in the overall process of NP delivery.^[^ ^107^, ^108^ ^]^ As discussed previously, the formation of the protein corona can profoundly alter the physicochemical identity of NPs, which in turn affects their blood pharmacokinetics, including circulation time and organ‐specific accumulation. ML provides a promising avenue to model and predict these complex in vivo behaviors, offering insights that extend beyond traditional experimental approaches.^[^ ^53^, ^57^, ^109^, ^110^ ^]^ For example, a recent study introduced dendPoint, an ML‐based in silico platform designed to predict the intravenous pharmacokinetics of dendrimers based on their structural and physicochemical features. This model accurately estimated parameters such as half‐life, clearance, volume of distribution, and dose recovery in the liver and urine, enabling informed dendrimer design prior to extensive animal testing.^[^ ^109^ ^]^ In another study, Lazarovits et al. developed a method to predict the biological behavior of NPs using a supervised DNN.^[^ ^57^ ^]^ They extracted NPs from the bloodstream at various time points, isolated the surface‐bound proteins, and analyzed them using proteomic mass spectrometry. The resulting protein profiles were used as input data for the model, while the NPs' clearance from the blood and accumulation in different organs served as output data to train the network. The neural network was tested by predicting the accumulation of NPs in the spleen and liver, achieving an accuracy greater than 94%. The model revealed that the mechanism behind NP uptake in these organs is driven by distinct patterns of proteins adsorbed on the surface of the NPs.^[^ ^57^ ^]^ Overall, understanding NP–protein interactions is critical for the successful design and application of NPs in biomedical contexts, particularly given their profound influence on blood circulation and systemic pharmacokinetics. The incorporation of ML techniques offers a powerful means to decode these complex nano–bio interactions. By leveraging data‐driven models, researchers can not only optimize NP design but also better predict in vivo behavior such as circulation time and organ accumulation,^[^ ^53^, ^57^ ^]^ ultimately accelerating the development of safer and more effective nanomedicines.

## 4 Extravasation to the TME

Extravasation of NPs into the TME is a complex process influenced by several factors, including the aberrant tumor vasculature, the perivascular TME, and the properties of the NPs themselves.^[^ ^111^ ^]^ The metabolic demands of rapidly dividing cancer cells create abnormal and leaky neovasculature, providing an opportunity for NPs to extravasate into the TME.^[^ ^112^, ^113^, ^114^, ^115^, ^116^ ^]^ However, the exact contribution of various tumor vascular segments to permeability remains unclear and complex.^[^ ^27^, ^117^, ^118^ ^]^ Moreover, recent studies have shown that NPs enter tumor tissues through an active transendothelial transport mechanism instead of passive extravasation.^[^ ^111^, ^119^, ^120^, ^121^ ^]^ This makes it challenging to understand the process of NP extravasation, necessitating a deeper exploration of the underlying mechanisms.

**Figure 6:**
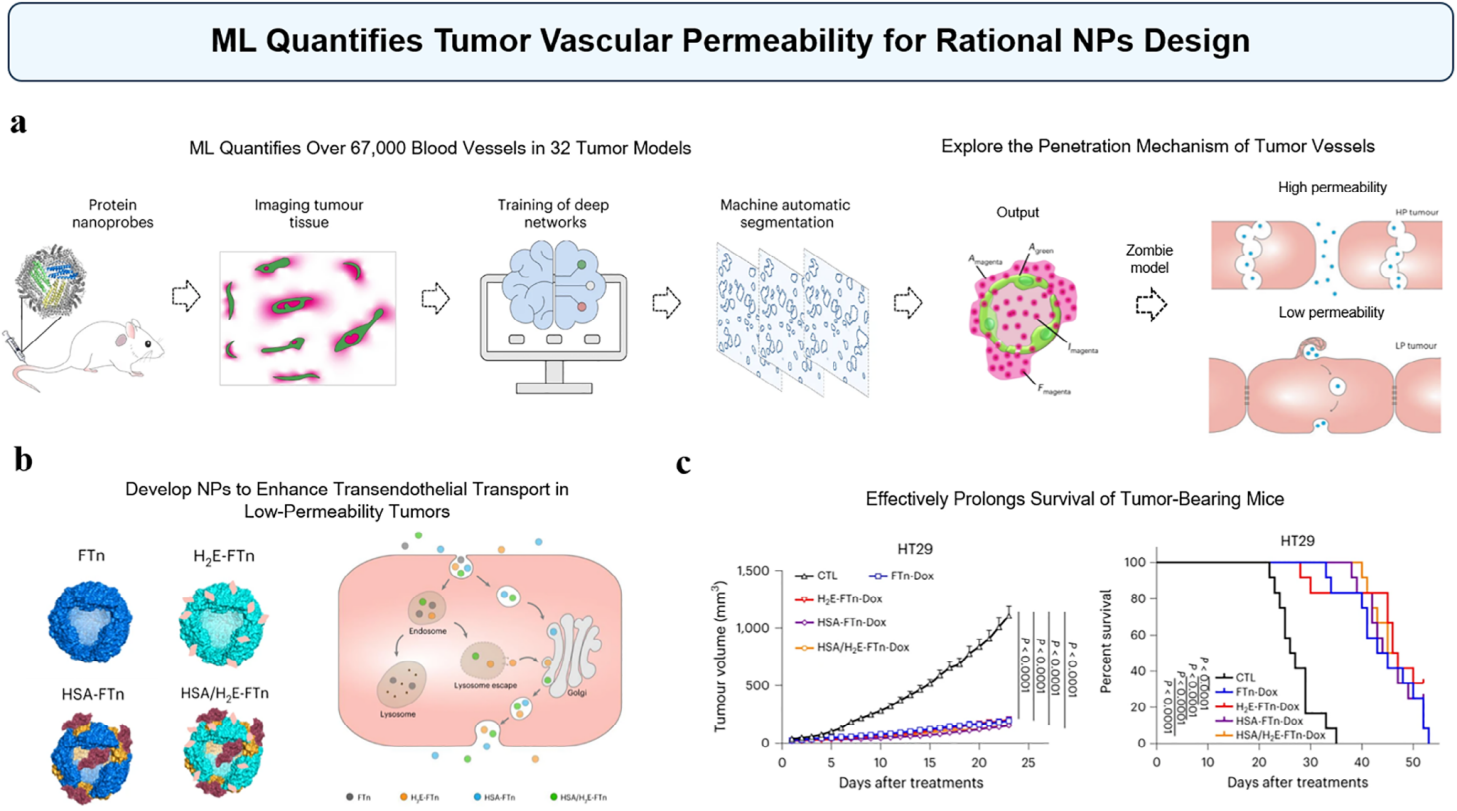
ML quantifies tumor vascular permeability for rational NPs design. a) The ML‐based image segmentation models were developed by training on annotated images of vessel and protein nanoprobe distributions in tumor tissues. The extracted tumor vascular features were then used to quantify the permeability of the vessels for subsequent analysis. The rightmost diagram shows high permeability tumors using passive extravasation and low permeability tumors using active transendothelial transport. b) Left: Diagrammatic representations of ferritin nanocages (FTn) and its variants. Right: Diagram illustrating a strategy that enhances transcytosis in endothelial cells by stimulating Golgi‐dependent exocytosis. c) Left: Tumor growth curves in tumor‐bearing mice with various treatments. Right: Kaplan–Meier survival curve of treated tumor‐bearing mice. Reproduced with permission.^[^ ^46^ ^]^ Copyright 2023, Springer Nature.

ML can be used to segment and quantify biological structures within the TME, such as tumor vasculature, cell nuclei, cancer cells, and phagocytic cells like Tumor‐Associated Macrophages (TAMs), revealing how NPs interact with and penetrate the TME. Recently, Zhu et al. have developed an ML‐assisted single‐vessel analysis method to investigate NP permeability in tumor vasculature (**Figure** **6**).^[^ ^46^ ^]^ This novel approach combined protein‐based nanoprobes with image segmentation‐based machine learning (nano‐ISML) to analyze over 67000 individual blood vessels from 32 tumor models. Researchers used Cy5.5‐labeled ferritin nanocages (FTn), which were administered to tumor‐bearing mice for in vivo imaging. Then two ML models were trained to automatically segment blood vessels and NP coverage areas in confocal images of tumor tissues. After segmentation, various features and parameters were extracted and computed for the following analysis. The study revealed significant heterogeneity in vascular permeability among different tumors and within individual tumors. The analysis demonstrated that NP delivery is primarily governed by passive extravasation in high‐permeability vessels and active transendothelial transport in low‐permeability vessels (Figure 6a). This finding challenges the traditional belief that enhanced leakiness is the dominant mechanism for NP delivery in tumors. The researchers then genetically tailored FTn to improve their permeability in low‐permeability tumors, which effectively prolonged the survival of tumor‐bearing mice (Figure 6b,c). This method provides valuable insights for designing effective nanomedicines and tailoring delivery strategies based on tumor‐specific vascular characteristics.^[^ ^46^ ^]^ In conclusion, understanding the factors that influence NP extravasation into the TME, particularly through the use of ML, is crucial for designing targeted and efficient nanomedicines. The ability to tailor NP properties based on specific vascular characteristics of tumors can significantly enhance the precision and effectiveness of cancer treatments.

## 5 Tumor Penetration and NP Distribution

After NPs extravasate into the TME, their ability to diffuse deeply and uniformly throughout the tumor tissue is critical for achieving therapeutic efficacy. However, the diffusion and penetration of NPs are significantly influenced by various factors, including blood vessel density, the extracellular matrix, interstitial fluid pressure, and the density of stromal cells such as TAMs.^[^ ^16^, ^30^, ^32^, ^122^, ^123^, ^124^, ^125^, ^126^ ^]^ These physiological features vary between tumors and within different regions of the same tumor. Understanding the relationship between tumor heterogeneity and NP accumulation and distribution is essential for optimizing nanomedicine design. By elucidating these interactions, researchers can develop strategies to improve NP penetration and efficacy.

**Figure 7:**
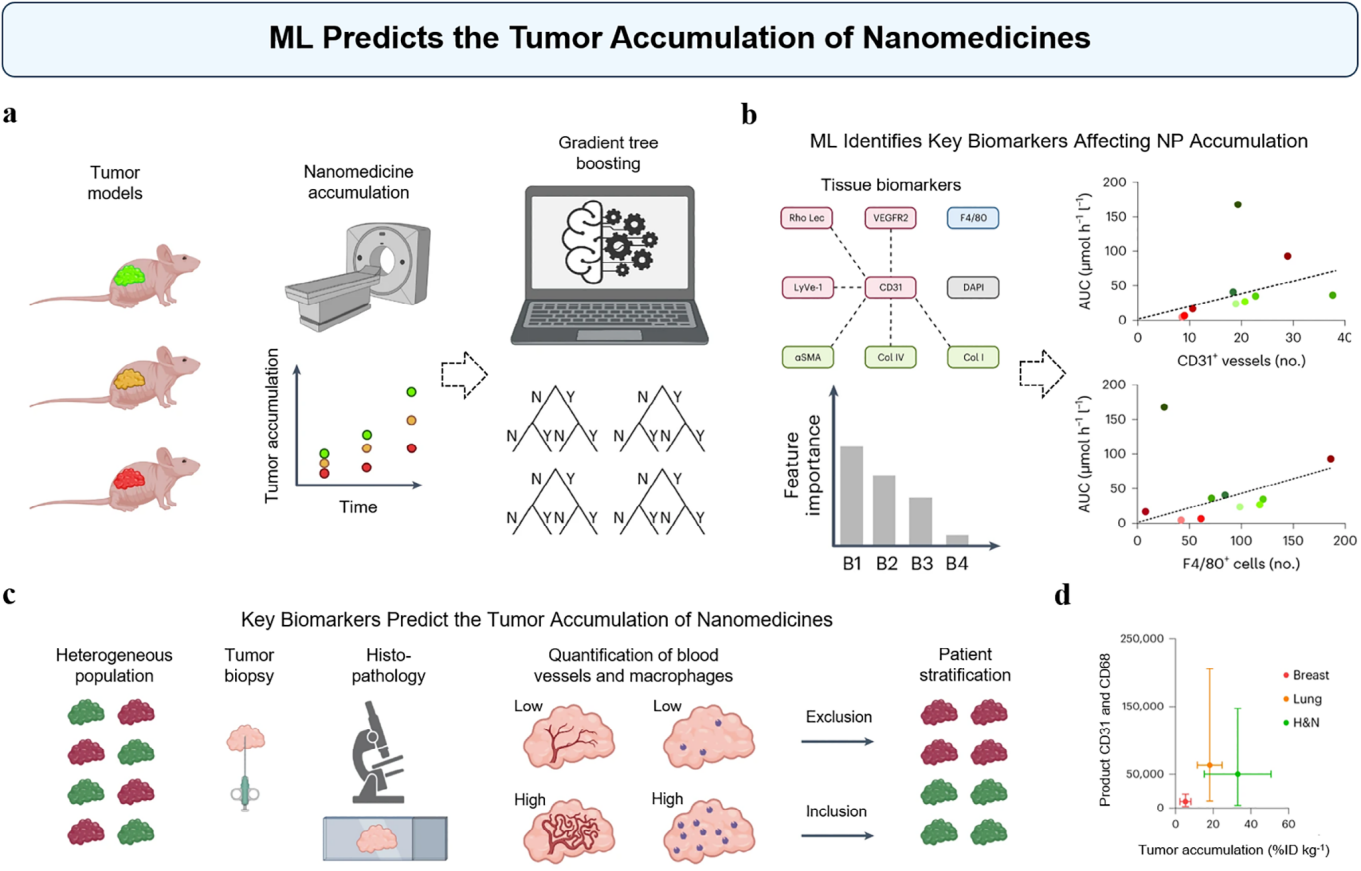
ML predicts the tumor accumulation of nanomedicines. a) Schematic of the experimental protocol to identify tumor‐tissue biomarkers correlating with nanomedicine accumulation in tumors. Tumor accumulation of PHPMA, a prototypic polymeric nanocarrier, was assessed using computed tomography–fluorescence molecular tomography in three mouse models with varying tumor targeting. b) Correlation analyses were performed using 23 TME features related to vasculature (red), stroma (green), macrophages (blue), and cellular density (grey). Gradient Tree Boosting‐based ML ranked feature importance, identifying blood vessel and Tumor‐Associated Macrophages (TAMs) densities as key predictive features. The rightmost two images show the correlation of these densities with total liposomal DXR tumor accumulation. c) Histopathological biomarker product score workflow for predicting nanomedicine tumor targeting. (d) Means of blood vessel and TAM product scores plotted against means of liposome tumor targeting, demonstrating that biomarker product scoring effectively identifies breast cancers as tumors with low nanomedicine accumulation. Reproduced under terms of the CC‐BY license.^[^ ^54^ ^]^ Copyright 2024, May et al., published by Springer Nature.

We can leverage ML to identify critical biomarkers by ranking feature importance and selecting those most influential to NP accumulation. Recently, May et al. have used ML to identify predictive biomarkers for the accumulation of nanomedicines in tumors (**Figure** **7**).^[^ ^54^ ^]^ The researchers employed ML techniques to analyze the interactions between NPs and various biological components within different tumor models. As shown in Figure 7a, they built a Gradient Tree Boosting model to predict nanomedicine accumulation across different tumor using 23 TME features. The ML process then focused on feature selection to determine which of these features were most predictive of nanomedicine accumulation. Ultimately, they identified blood vessel density and TAM density as key biomarkers with the help of ML models (Figure 7b). As exemplified in Figure 7b, there generally existed a good correlation between tumor blood vessel and TAM density and nanomedicine accumulation. However, the E35CR model was identified as a distinct outlier, exhibiting the highest accumulation of nanomedicine while having intermediate levels of CD31^+^ blood vessels and very low levels of F4/80^+^ TAM. The researchers did not provide detailed discussion or speculation on the possible reasons for this outlier. The identified biomarkers were further integrated into a histopathological biomarker product score, which was used to quantify the potential for effective nanomedicine delivery in different tumor types. According to the study, the biomarker scores correctly classified nine out of the ten tumor models as either high or low nanomedicine accumulators, with only the E35CR model being misclassified as a low accumulator (a false negative). To further validate the clinical applicability of the scoring system, the researchers analyzed patient tumor samples, focusing on breast, lung, and head and neck cancers. They quantified blood vessel and TAM density, correlating these with nanomedicine accumulation. The biomarker product scoring effectively identified breast cancers as tumors with low nanomedicine accumulation (Figure 7d). This knowledge is essential for patient stratification^[^ ^27^ ^]^ in clinical trials, accurately identifying patients who are more likely to benefit from nanomedicine treatments and optimizing therapeutic efficacy (Figure 7c).^[^ ^54^ ^]^

**Figure 8:**
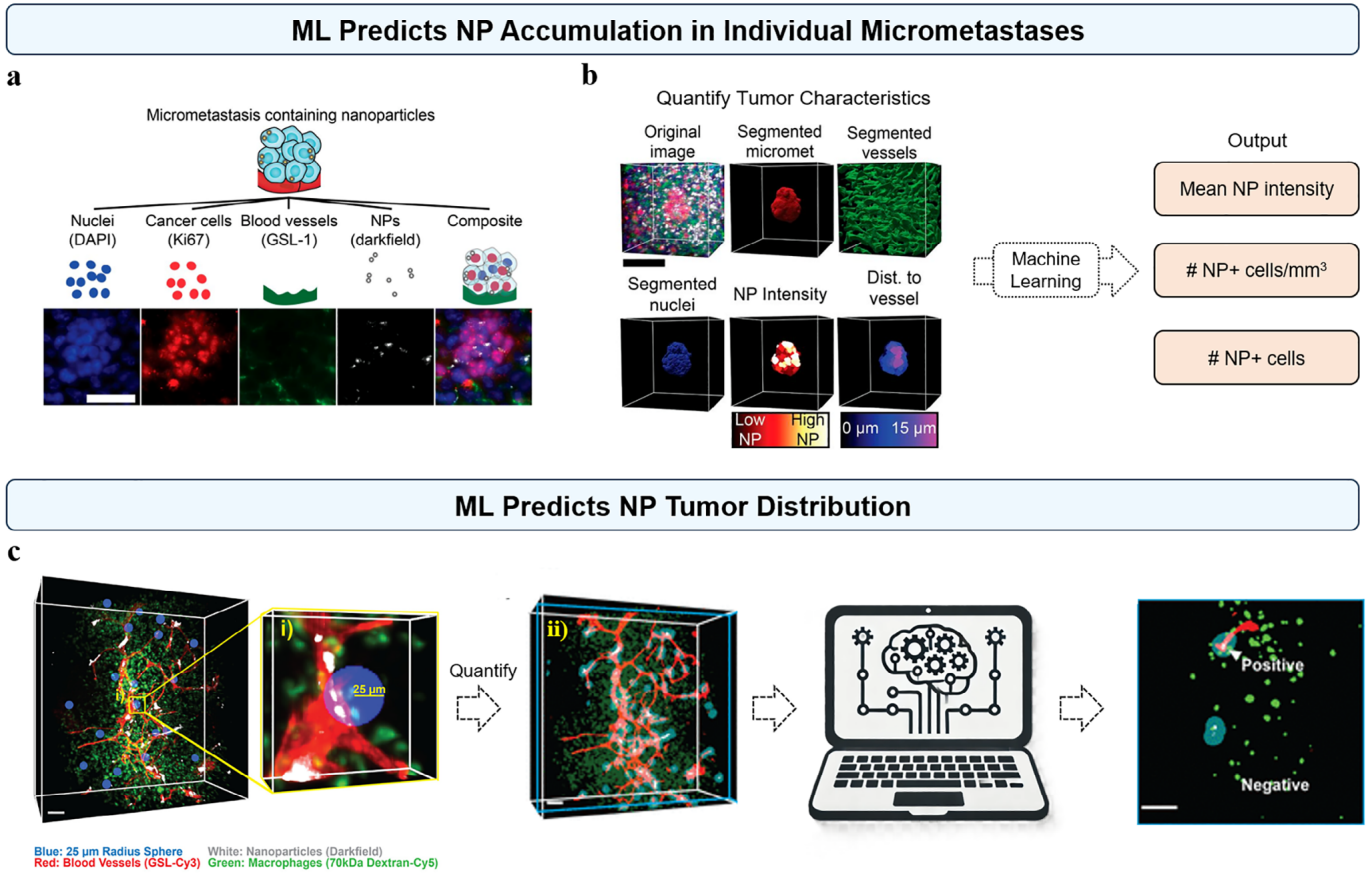
a,b) ML predicts NP accumulation in individual micrometastases. a) Light‐sheet imaging captured individual channels for nuclei (DAPI), cancer cells (Ki67), blood vessels (GSL‐1), and NPs (darkfield). b) ML image segmentation enabled detailed analysis of 3D microscopy images. A predictive model of NP delivery to micrometastases was created using 3D imaging data and physiological characteristics. c) ML predicts NP tumor distribution. The leftmost image shows a 3D U‐87 MG tumor with 25 µm radius sampling regions. (i) Close‐up of the tumor with a 25 µm radius sample region. (ii) 3D image with NP boundaries overlaid on tumor features: blue (25 µm radius sphere), white (NPs), red (blood vessels), green (macrophages). Data from blood vessels and macrophages were used to train a Logistic Regression model to classify 25 µm radius regions as NP‐positive or NP‐negative. a, b) Reproduced with permission.^[^ ^67^ ^]^ Copyright 2019, National Academy of Sciences. c) Reproduced with permission.^[^ ^70^ ^]^ Copyright 2023, American Chemical Society.

Researchers can also employ ML in conjunction with advanced imaging technologies to segment and quantify the pathophysiological features of tumor tissues, establishing an ML model to predict NP accumulation within tumors.^[^ ^67^, ^70^, ^127^ ^]^ In a recent study, Kingston et al. have utilized 3D microscopy combined with ML to predict NP delivery to individual micro‐metastases (**Figure** **8**a,b).^[^ ^67^ ^]^ By imaging micro‐metastases in the livers of mice and analyzing these images with ML and computational methods, the researchers created a high‐throughput image analysis workflow. This method allowed for the profiling of 1301 micro‐metastases, revealing that NPs accessed a higher proportion of cells in micro‐metastases compared to primary tumors due to their proximity to blood vessels and shorter diffusion distance. Then, they developed Support Vector Machine models to predict NP delivery based on the physiology of micro‐metastases, providing a valuable tool for targeting early metastatic growth more effectively (Figure 8b).^[^ ^46^ ^]^ While ML can be used to predict NP accumulation across different tumors, we may further utilize it to predict the specific spatial distribution of NPs within tumor tissues, providing a detailed understanding of their localization. Recently, MacMillan et al. have demonstrated the use of a Logistic Regression model to predict the distribution of NPs within heterogeneous tumor tissues (Figure 8c).^[^ ^70^ ^]^ Researchers developed a computational technique that correlated the spatial distribution of NPs with the patterns of biological structures within the tumor. Using a 25 µm spherical probe radius, the model achieved an 88% accuracy in predicting NP location within the tumor. This approach highlights the potential of ML to characterize tumor heterogeneity computationally and predict the distribution of NPs within tumor tissues, which could significantly enhance the precision of NP targeting and improve therapeutic efficacy.^[^ ^70^ ^]^ Meanwhile, another study used ML to conditionally generate pixel‐level intratumoral NP distribution images based on the spatial information of tumor vessels and cell nuclei, allowing for quantitative analysis and optimization of NP delivery in tumor tissues.^[^ ^128^ ^]^ Other studies focused on the overall delivery efficiency, utilizing ML to predict NP delivery to tumors and significantly enhancing the design of cancer nanomedicines.^[^ ^110^, ^129^, ^130^ ^]^ In summary, understanding tumor penetration and NP distribution through the use of ML offers a transformative approach to improving nanomedicine. By accurately predicting both accumulation and distribution of NPs within heterogeneous tumor tissues, these advanced computational methods pave the way for more targeted and effective cancer treatments.^[^ ^131^ ^]^

## 6 Cellular Internalization

Cellular internalization is the process by which cells absorb external particles, such as NPs, from their surrounding environment.^[^ ^132^, ^133^ ^]^ This process is crucial for the effectiveness of NP‐based therapies, as it determines the delivery and release of therapeutic agents within target cells, directly impacting therapeutic outcomes and the success of nanomedicines. The process of internalization is influenced by various factors, including the physicochemical properties of NPs, the biological environment, and the characteristics of the target cells.^[^ ^132^, ^134^, ^135^, ^136^ ^]^ Understanding how NPs are internalized by cells is essential for optimizing their design and enhancing their therapeutic efficacy. By gaining insights into the mechanisms of NP–cell interactions, researchers can tailor NPs to improve their uptake by specific cell types, thereby increasing the concentration of therapeutic agents delivered to target sites and minimizing off‐target effects.^[^ ^137^, ^138^, ^139^ ^]^

**Figure 9:**
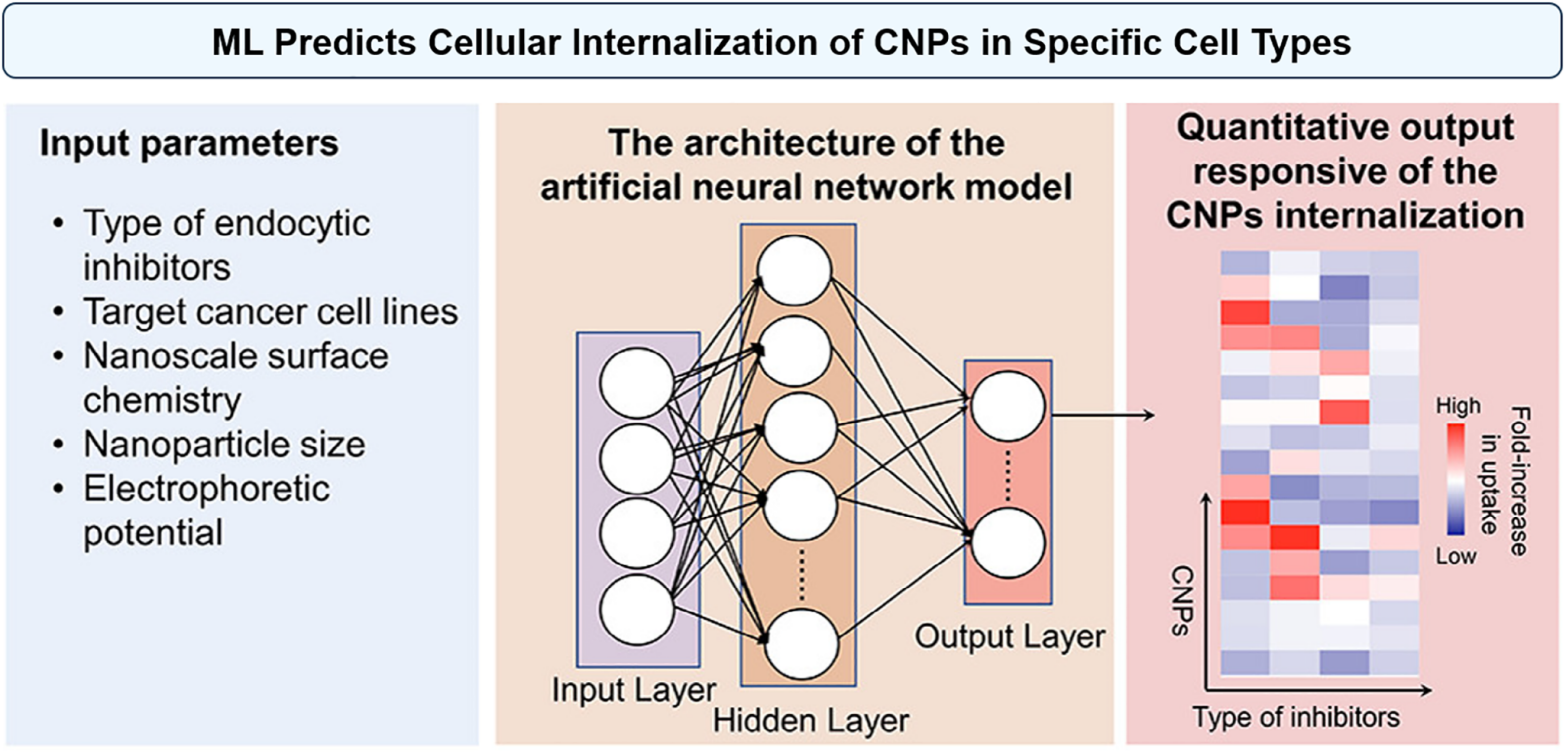
ML predicts cellular internalization of carbon nanoparticles (CNPs) in specific cell Types. An artificial neural network (ANN) model was trained using the design parameters of eight CNPs to predict their cellular internalization. The model utilized the particle size, zeta potential, and surface chemistry of the eight CNPs, along with the inhibitor and the targeted cell, as input parameters. It assigned a quantitative value to each CNP based on its cellular internalization via the specific endocytosis pathway. Reproduced with permission.^[^ ^58^ ^]^ Copyright 2020, American Chemical Society.

**Figure 10:**
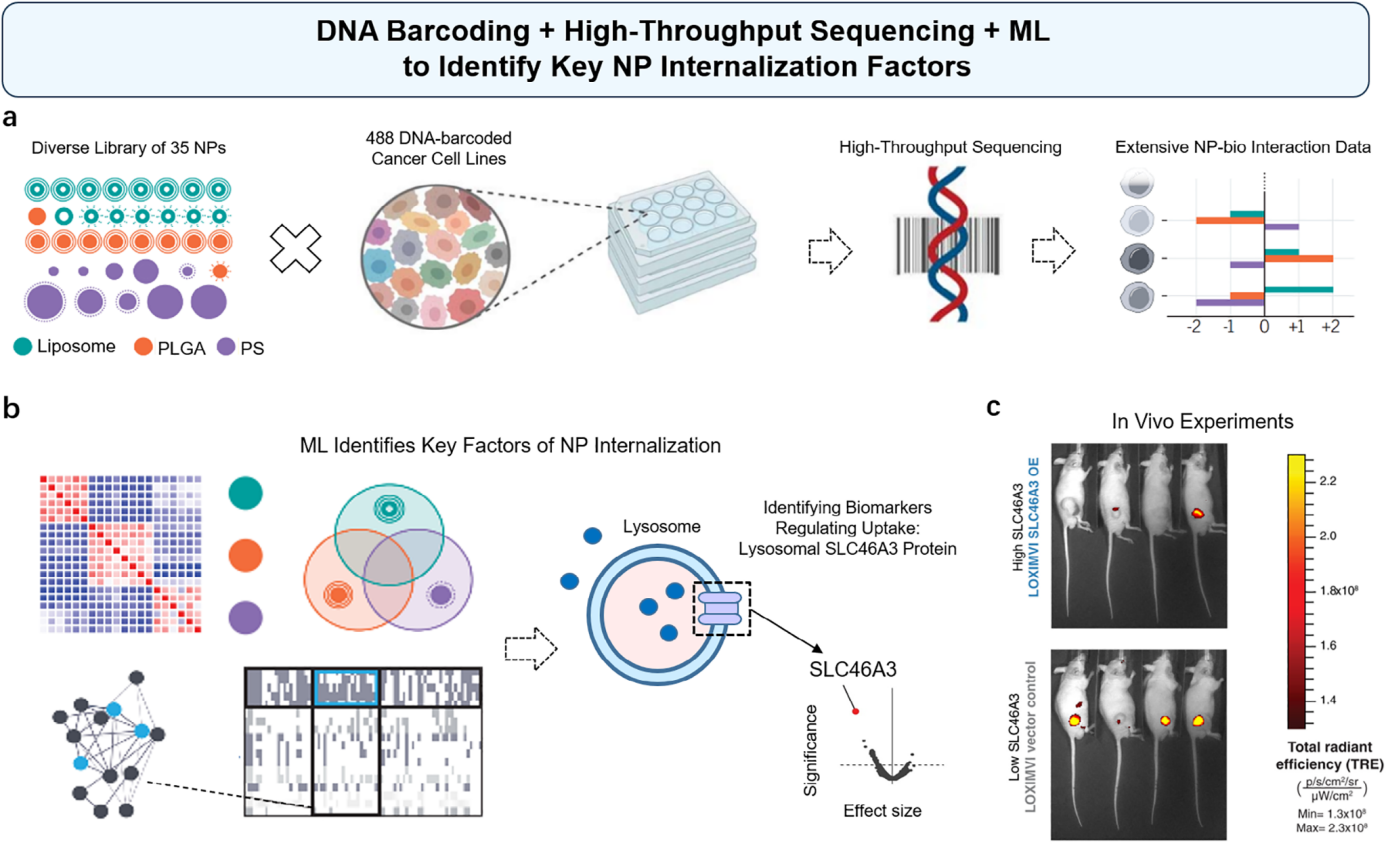
Using DNA barcoding, high‐throughput sequencing, and ML to identify key factors in NP internalization. a) A schematic of the nanoPRISM assay illustrates the incubation of fluorescently labeled NPs with pooled cancer cells, followed by Fluorescence‐Activated Cell Sorting and DNA barcode sequencing for analysis. b) Omics integration identified predictive biomarkers of cellular uptake. Native expression of the lysosomal transporter SLC46A3 was found to be a key factor in predicting NP–cell interaction. Univariate analysis and ML showed that SLC46A3 expression is strongly inversely correlated with liposome association. c) Whole‐animal fluorescence images were captured 24 h after intratumoral injection of LIPO‐0.3% PEG* NPs in mice with low and high SLC46A3 expression. Reproduced with permission.^[^ ^45^ ^]^ Copyright 2022, American Association for the Advancement of Science.

ML models can be developed to predict NP uptake based on their physicochemical properties and the specific cancer cells involved.^[^ ^45^, ^58^, ^97^, ^98^, ^140^, ^141^, ^142^, ^143^, ^144^, ^145^ ^]^ In a recent study, Alafeef et al. have applied ML to estimate the internalization behavior of carbon nanoparticles (CNPs) in different breast cancer cell lines (**Figure** **9**).^[^ ^58^ ^]^ Using a high‐throughput screening protocol, the researchers generated data on NP uptake by measuring the fold increase in cell viability after blocking specific endocytosis pathways with inhibitors, which was then used to train an Artificial Neural Network (ANN) model. The model predicted cellular internalization patterns based on NP size, zeta potential, surface chemistry, type of inhibitor, and targeted cell type. Then, they attempted to minimize the experimental data required for training the ANN model. Specifically, the ANN model achieved a high predictive performance (Q^2^ = 0.9030) when trained on a randomly selected subset of 100 data points, which represented 20.83% of the total dataset. This highlights the model's ability to accurately predict NP internalization using only a fraction of the experimental data, significantly reducing the need for extensive laboratory work.^[^ ^58^ ^]^ Beyond predicting cellular internalization, we can also apply ML to analyze multi‐omics data to reveal relationships between genes, proteins, and other biomarkers with NP uptake, identifying key factors affecting NP internalization.^[^ ^45^, ^146^, ^147^ ^]^ In a recent study, Boehnke et al. have combined massively parallel pooled screening with ML to investigate the genomic determinants of NP delivery (**Figure** **10**).^[^ ^45^ ^]^ As shown in Figure 10a, the researchers developed an NP library of 35 formulations and conducted a high‐throughput screen across 488 DNA‐barcoded cancer cell lines to explore NP–cell interaction profiles. ML algorithms were then used to analyze multi‐omics data, identifying predictive biomarkers for NP uptake and constructing genomic NP trafficking networks. Specifically, the study highlights the discovery of SLC46A3, a solute carrier (SLC) transporter localized to the lysosome, as a negative regulator of liposomal NP uptake, which could influence NP accumulation and delivery in cancer cells. Although the biological function of SLC46A3 in cancer is not yet fully understood, its reported relationship with lipid catabolism likely explains its strong influence on liposomal NP uptake (Figure 10b). This finding was validated in vitro using cancer cell lines and in vivo using mouse models, demonstrating the gene's crucial regulatory role (Figure 10c). Modulating the expression of this gene might increase the efficiency of liposomal NP uptake, enhancing the delivery of drugs to cancer cells. This study demonstrates the power of integrated genomic and computational approaches to uncover the biological mechanisms underlying NP cellular uptake, facilitating the rational design of nanoformulations.^[^ ^45^ ^]^

**Figure 11:**
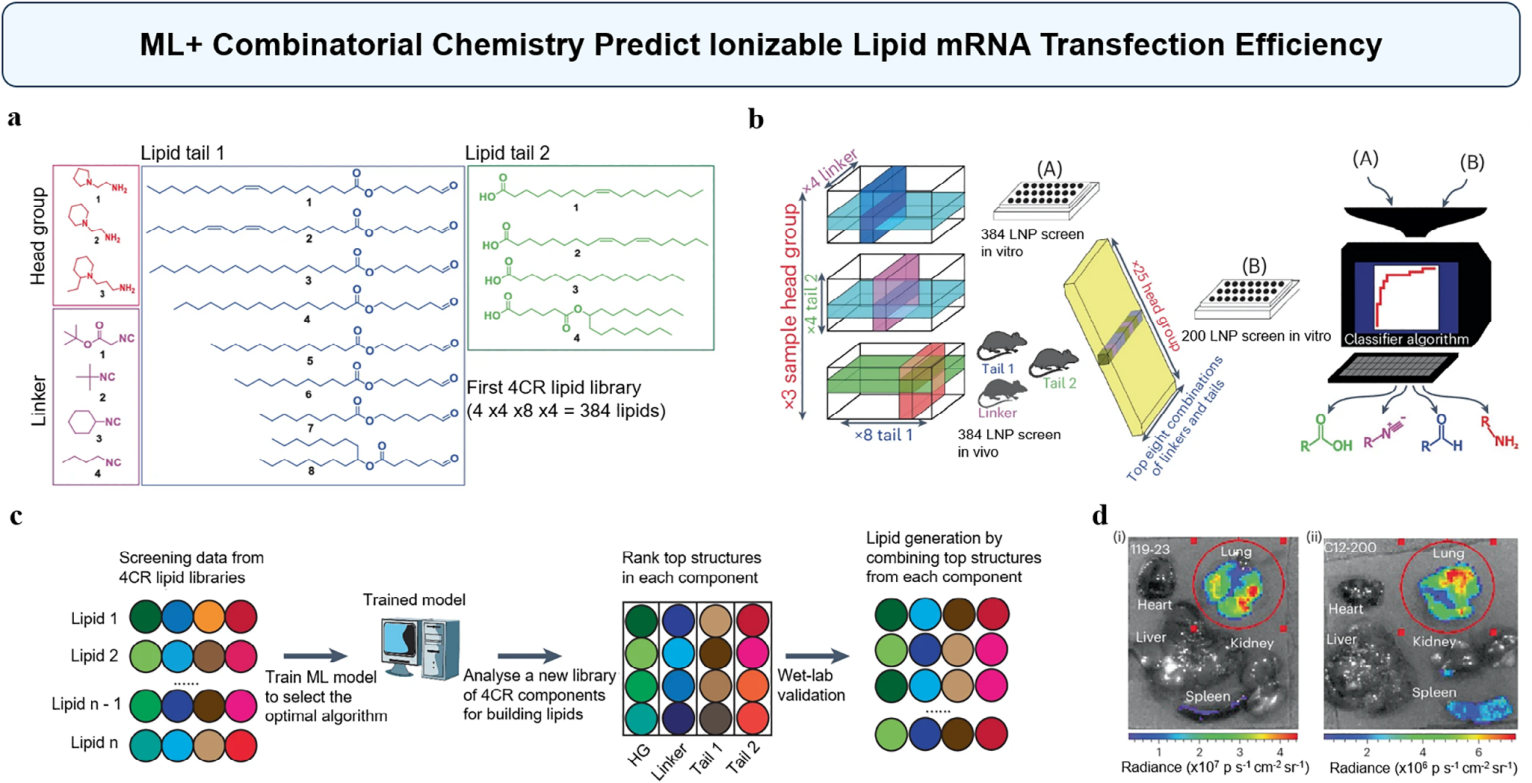
Using ML and combinatorial chemistry to predict ionizable lipid mRNA transfection efficiency. a) Structures of chemical compounds in four components used as 4CR Ugi reactants for creating a combinatorial library of 384 ionizable lipids. b) A schematic diagram illustrating the process of synthesizing and screening a lipid library to generate data for training ML models. c) ML ranked the top structures in each component, followed by wet‐lab validation of lipids using these top structures. d) This figure shows representative In Vivo Imaging System images of mouse organs taken 6 h after intravenous administration of lung‐targeted mLuc lipid nanoparticles (LNPs) containing 119‐23 and C12‐200 (0.25 mg mRNA per kg mouse). Region of Interest 1 demonstrates signal intensities of 3.129 × 10^8^ (i) and 5.319 × 10^7^ (ii). Reproduced with permission.^[^ ^47^ ^]^ Copyright 2024, Springer Nature.

Additionally, we can combine ML with high‐throughput experiments, where the resulting data can train ML models to efficiently screen large libraries and identify optimal candidate molecules for subsequent experimental validation. Recently, Li et al. have combined ML with combinatorial chemistry to accelerate the discovery of ionizable lipids for mRNA delivery (**Figure** **11**).^[^ ^47^ ^]^ As shown in Figure 11b, researchers created a library of 584 ionizable lipids and used their mRNA transfection data to train ML models. The best‐performing model was applied to screen a virtual library of 40000 lipids, identifying several high‐performing candidates (Figure 11c). One lipid, 119‐23, showed exceptional mRNA delivery efficiency in muscle and immune cells. When formulated for lung‐targeted delivery, 119‐23 achieved a fivefold enhancement in transfection potency compared with other lipid nanoparticles (LNPs), demonstrating significant potential for applications in vaccines and immunotherapies (Figure 11d). This approach not only speeds up the discovery process but also enhances the precision of mRNA delivery systems, potentially leading to more effective therapies.^[^ ^47^ ^]^ Overall, integrating ML and high‐throughput screening enables accurate predictions and enhancements of NP uptake, paving the way for the development of highly effective and targeted therapies that improve patient outcomes.^[^ ^47^, ^148^, ^149^, ^150^ ^]^

## 7 Bioactivity Assessment & Therapeutic Efficacy

To achieve optimal therapeutic outcomes, NPs must exhibit high bioactivity and therapeutic efficacy once delivered to target cells. High bioactivity ensures that NPs can effectively interact with target cells to induce desired therapeutic effects while minimizing toxicity to normal cells.^[^ ^12^, ^151^, ^152^, ^153^, ^154^, ^155^ ^]^ This balance between efficacy and safety is crucial for the successful application of nanomedicine in clinical settings.

**Figure 12:**
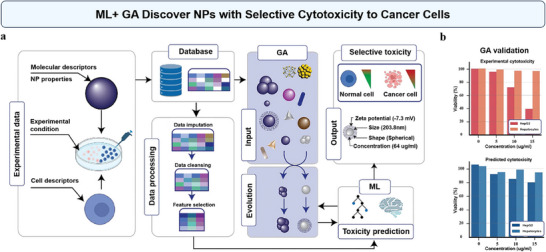
Using ML and Genetic Algorithm (GA) to discover NPs with selective cytotoxicity to cancer cells. a) Schematic depicting the screening of selectively cytotoxic NPs using an ML‐reinforced GA. b) ML‐reinforced GA was validated by reproducing experimental results of selective cytotoxicity of ZnO NPs on HepG2 and hepatocyte cell lines. Reproduced with permission.^[^ ^156^ ^]^ Copyright 2023, Wiley‐VCH.

**Figure 13:**
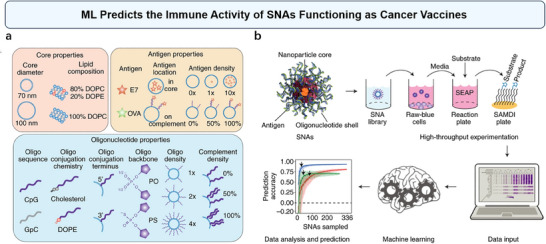
ML predicts the immune activity of spherical nucleic acids (SNAs) functioning as cancer vaccines. a) The 11 structural parameters, organized by core, antigen, and oligonucleotide property categories, resulted in a potential design space of 3072 variants. DOPC, 1,2‐dioleoyl‐sn‐glycero‐3‐phosphocholine; DOPE, 1,2‐dioleoyl‐sn‐glycero‐3‐phosphoethanolamine; PO, phosphodiester; PS, phosphorothioate. b) Following the creation of a library of SNA‐based cancer vaccines, high‐throughput mass spectrometry was employed to characterize their cellular immune activity. These data were subsequently used to train an ML algorithm for predicting the immune activity of each SNA in the library. Reproduced with permission.^[^ ^159^ ^]^ Copyright 2019, Springer Nature.

We can integrate ML with optimization algorithms such as Genetic Algorithms (GA) to refine NP properties and treatment parameters, thereby maximizing therapeutic efficacy while minimizing dosage.^[^ ^156^, ^157^ ^]^ In a recent study, Jyakhwo et al. have employed an ML‐reinforced GA to discover inorganic NPs with selective cytotoxicity toward cancer cells (**Figure** **12**).^[^ ^156^ ^]^ In the first step, a Gradient Boosting Regression model was built to predict the viability of NP‐treated cell lines, achieving high predictive accuracy. Then, they employed GA, a general optimization technique inspired by natural selection, to rapidly generate and evaluate a large number of in silico candidate NPs, iteratively evolving their properties to identify optimal solutions exhibiting highest selective cytotoxicity (Figure 12a). The study identified AgNPs with a fitness score of 42% against HepG2 liver cancer cells, while exhibiting minimal toxicity toward normal hepatocytes. The model's predictions were compared with experimental results from other studies, showing that it could reproduce cytotoxicity patterns for different NPs and cell lines (Figure 12b). This approach significantly reduces the need for extensive, time‐consuming experimental testing, potentially accelerating the development of more effective and safer NP‐based drug delivery systems for cancer treatment.^[^ ^156^ ^]^ On the other hand, bioactivity prediction models have also been built with the use of ML. Recently, Yamankurt et al. have developed a high‐throughput method combined with ML to evaluate the cellular immune responses of spherical nucleic acids (SNAs) functioning as cancer vaccines (**Figure** **13**).^[^ ^68^ ^]^ As shown in Figure 13a, they synthesized approximately 1000 SNAs with different architectures by adjusting 11 design parameters. Using high‐throughput mass spectrometry, they measured the enzymatic activity from over 17000 SAMDI (self‐assembled monolayers for MALDI, where MALDI stands for matrix‐assisted laser desorption/ionization) spectra and trained a supervised ML algorithm to predict immune activity based on SNA structural features (Figure 13b). This method enables the rapid screening of SNAs to identify those with the highest immune activation potential, highlighting the capability of ML to accelerate the discovery of nanomedicines with superior bioactivity and therapeutic efficacy.^[^ ^68^ ^]^ Beyond focusing on the bioactivity of NPs, ML can be applied on a broader scale. For instance, ML can be employed to analyze large‐scale clinical datasets, enabling researchers to identify the physicochemical properties of NPs and therapeutic strategies that most significantly impact clinical outcomes.^[^ ^55^ ^]^ In summary, integrating ML into the assessment of NP bioactivity and therapeutic efficacy accelerates the discovery of effective therapies with reduced side effects.^[^ ^158^ ^]^ As evidenced by recent studies, ML enables rapid screening and selection of NP candidates with superior bioactivity, paving the way for more targeted and efficient therapies that hold promise for improving patient outcomes in diverse medical contexts.^[^ ^159^ ^]^

## 8 Challenges and Prospects

In the preceding sections, we highlighted the transformative potential of ML in NP design for precision cancer drug delivery. From accelerating NP synthesis to predicting complex nano–bio interactions and optimizing therapeutic efficacy, ML has demonstrated substantial promise across multiple stages of the nanomedicine pipeline. However, despite these advancements, several critical challenges remain. Limitations such as insufficient and biased datasets, lack of informative nanodescriptors, and limited model interpretability continue to hinder the full integration of ML into clinical nanomedicine. Addressing these issues is essential to realize the full potential of ML‐driven NP design in cancer therapy.

### 8.1 Data Size and Data Bias

One of the most pressing challenges in applying ML to nanomedicine lies in the limited size and quality of available datasets. Many current ML studies in this field rely on relatively small datasets, often comprising only hundreds to a few thousand data points. Such data volumes are insufficient for training complex ML models, particularly deep learning architectures, which typically require large‐scale datasets to achieve robust generalization and avoid overfitting.^[^ ^49^, ^50^, ^137^, ^160^ ^]^ In contrast, recent breakthroughs in protein science have been enabled by the availability of massive, high‐quality datasets and publicly accessible repositories, which support the development of highly generalizable models and advancement of protein engineering.^[^ ^161^, ^162^, ^163^ ^]^

The situation in nanomedicine is uniquely challenging. The field encompasses a vast diversity of NP types and design parameters, and generating high‐quality experimental data is labor‐intensive, time‐consuming, and costly.^[^ ^12^, ^19^, ^164^, ^165^ ^]^ These practical constraints make it difficult to construct comprehensive and structured nanomedicine databases.^[^ ^164^, ^165^ ^]^ As a result, many current ML models in this domain may suffer from overfitting due to limited training data. In the short term, researchers can enhance model performance on small datasets by improving data quality, employing sampling strategies such as active learning, and applying techniques to prevent overfitting.^[^ ^59^, ^164^, ^166^ ^]^ In the long term, however, the development of large‐scale, high‐quality nanomedicine datasets is indispensable. Emerging experimental techniques, such as high‐throughput screening,^[^ ^43^, ^44^ ^]^ microfluidics,^[^ ^24^, ^77^ ^]^ and DNA barcoding,^[^ ^167^, ^168^, ^169^ ^]^ offer promising avenues for generating vast quantities of NP data more efficiently. Notably, recent efforts in autonomous and ML‐assisted experimentation, such as the development of ML‐enabled chemical synthesis robots for nanomaterial optimization,^[^ ^62^, ^64^ ^]^ illustrate the potential of automation in scaling data generation. Another complementary strategy is literature mining,^[^ ^52^, ^53^, ^55^ ^]^ which can extract NP‐related data from published studies to help build comprehensive datasets. However, such data often suffer from inconsistencies due to lab‐to‐lab variability in experimental protocols, instrumentation, and operator expertise, and may have limited accessibility.^[^ ^170^ ^]^ To address these issues, it is advocated that standardized experimental procedures, transparent data sharing policies, and community‐driven initiatives be established to promote openness, reproducibility, and interoperability in nanomedicine data resources. In addition, data‐centric strategies inspired by computer vision and other domains, such as data augmentation^[^ ^36^, ^171^ ^]^ and transfer learning,^[^ ^172^, ^173^ ^]^ hold potential to alleviate data scarcity in nanomedicine. For instance, ML model can generate realistic intratumoral NP distribution images that capture spatial relationships and enable virtual analysis, potentially expanding datasets to enhance model robustness and address data scarcity in nanomedicine.^[^ ^128^ ^]^ However, the applicability and reliability of these approaches in the context of complex nano–bio systems remain to be fully validated, and further research is required to assess their feasibility and limitations.

Beyond the challenge of small dataset sizes, data bias presents another significant obstacle in ML applications. Data bias occurs when the training data fails to adequately represent the target population, resulting in models that perform poorly in certain scenarios. Many current studies implicitly encounter this issue; for instance, datasets may predominantly include specific tumor types, underrepresenting rare variants, be limited to particular NP characteristics, or exhibit an imbalance between positive and negative samples.^[^ ^25^, ^164^ ^]^ Such biases can limit the model's ability to make accurate predictions on new or varied data, reducing its reliability across different experimental or biological conditions. To address this, researchers can adopt strategies aimed at enhancing data representativeness, balancing sample distributions, and improving model adaptability, thereby mitigating the impact of bias on performance and ensuring more reliable outcomes in real‐world applications.^[^ ^164^, ^174^, ^175^ ^]^

### 8.2 Data Representation

A key challenge in applying ML to NP design for cancer drug delivery is the representation of NP. Unlike small molecules and proteins, which benefit from relatively standardized and widely accepted representation methods such as Simplified Molecular Input Line Entry System strings or amino acid sequences,^[^ ^176^, ^177^ ^]^ NPs exhibit significant structural and functional diversity. Various types of NPs, such as liposomes, polymeric NPs, and metallic NPs, have distinct characteristics that require specific sets of descriptors.^[^ ^19^, ^178^ ^]^ This diversity complicates the development of a unified or standardized representation framework.

Furthermore, nanomedicine data are inherently multimodal, encompassing a range of formats, including microscopic images, spectroscopic data, physicochemical parameters.^[^ ^59^, ^67^, ^68^, ^70^ ^]^ Integrating these diverse data modalities into a cohesive, machine‐readable form presents a significant challenge. In addition to this, most studies rely on descriptors that characterize the NP's material properties, but these representations often lack biological relevance, failing to capture the complex interactions between NPs and biological systems. Developing informative descriptors that reflect interactions with proteins, cell membranes, immune components, or metabolic networks could enhance the predictive power of ML models. For example, Reker et al. introduced MD‐derived descriptors that more accurately characterize the interactions between drug molecules and excipients, enhancing the prediction of self‐assembling NP formation.^[^ ^44^ ^]^ As another example, Duan et al. proposed a novel biologically informed descriptor, fluorescence change (FC), derived from fluorescamine labeling of proteins in the presence or absence of engineered nanomaterials (ENMs). This FC metric reflects protein–ENM interaction dynamics and was used to train RF models for predicting protein corona composition. Their results demonstrated that models using FC significantly outperformed those based on conventional physicochemical descriptors, especially for diverse ENMs such as metal oxides, nanocellulose, and 2D materials, highlighting the value of biologically responsive descriptors in improving ML predictions.^[^ ^101^ ^]^ Future research should focus on developing NP representations that integrate both material properties and biological interactions to enhance ML model accuracy in nanomedicine.

### 8.3 Model Interpretability

Model interpretability represents another major challenge that hinders the clinical translation of ML in nanomedicine.^[^ ^179^, ^180^ ^]^ Importantly, the degree of interpretability is closely tied to the type of model employed. Many current studies in the field tend to favor tree‐based approaches, such as RF, which are relatively more interpretable.^[^ ^47^, ^53^, ^54^ ^]^ In these models, the contribution of each feature can be quantified through feature importance scores, enabling researchers to gain insights into the underlying biological mechanisms. Several studies have leveraged this interpretability to extract meaningful biological knowledge.^[^ ^45^, ^69^ ^]^ For instance, Boehnke et al. employed univariate analysis in conjunction with RF‐based feature importance ranking and identified SLC46A3 as a negative regulator of liposomal NP uptake. In addition, combining interpretability frameworks with tools from other domains, such as network science, can further aid nanoscientists in understanding ML models. For example, Yu et al. introduced a tree‐based framework that integrates feature importance with interaction network analysis to predict pulmonary immune responses and lung burden of NPs. This approach revealed complex dependencies between NP properties and biological outcomes, enhancing interpretability and offering insights for NP design, even with small, heterogeneous datasets.^[^ ^53^ ^]^

However, for more complex deep learning architectures, interpretability remains an open challenge and continues to be an active area of ML research.^[^ ^181^, ^182^ ^]^ Unlike tree‐based models, deep learning models are typically less transparent, complicating the interpretation of their predictions. While early efforts to interpret these models often focused on correlation‐based explanations, recent research has shifted toward uncovering causal and mechanistic insights.^[^ ^183^ ^]^ Looking forward, next‐generation models should integrate human reasoning capabilities and domain expertise to deliver meaningful and actionable explanations. Such hybrid approaches will be essential for earning biomedical trust, meeting regulatory standards, and enabling safe, effective deployment of ML in nanomedicine.^[^ ^40^, ^48^, ^184^ ^]^

## 9 Conclusion

In traditional nanomedicine research, the workflow predominantly follows a standard methodology: synthesis, formulation, and characterization of nanomedicine candidates, followed by in vitro proof‐of‐concept studies and in vivo antitumor animal testing. This conventional approach is inherently time‐consuming and inefficient, often requiring extensive trial‐and‐error experiments that demand substantial time and resources. The emergence of ML has profoundly transformed traditional cancer nanomedicine, infusing it with powerful capabilities. Nanomedicine delivery systems are intrinsically complex, involving numerous variables from synthesis to nano–bio interactions. ML is highly proficient at navigating vast parameter spaces to find optimal combinations for target properties, modeling intricate relationships to predict complex nano–bio interactions, and identifying patterns and key factors. Therefore, the integration of ML into NP drug delivery can accelerate the development process and facilitate the rational design of nanomedicines. In this review, we summarize how ML can be leveraged to enhance nanomedicine across all stages of the NP delivery system, including NP synthesis and formulation, NP–protein interactions, blood circulation, extravasation to the TME, tumor penetration and NP distribution, and cellular internalization. We also discuss recent notable studies to highlight the advancements and ongoing challenges in the field. Our aim is to highlight how ML can enhance each of these stages associated with precision drug delivery, offering valuable insights and practical guidance for scientists in their pursuit of developing more effective and targeted nanomedicine therapies.

Since the early 1990s, NP‐based drug delivery systems have made significant inroads into clinical practice, with various platforms, such as polymeric NPs, lipid‐based NPs, and inorganic NPs, already approved by FDA.^[^ ^6^, ^185^, ^186^ ^]^ Notably, the recent success of LNPs in COVID‐19 mRNA vaccines,^[^ ^187^, ^188^, ^189^ ^]^ such as mRNA‐1273 (Moderna)^[^ ^190^ ^]^ and BNT162b2 (Pfizer‐BioNTech),^[^ ^191^ ^]^ highlights the clinical potential of nanomedicine. As the field progresses, ongoing clinical trials are shifting from conventional drug delivery systems to more advanced platforms that integrate active targeting, stimuli‐responsive mechanisms, and nucleic acid‐based therapies. These complex designs introduce numerous tunable parameters, vastly expanding the design space. In this context, ML offers a powerful approach to guide rational NP development by efficiently navigating this multidimensional landscape.^[^ ^47^, ^68^ ^]^ Looking ahead, the next‐generation NP platforms will likely emerge from an interdisciplinary convergence of ML with a suite of cutting‐edge technologies such as high‐throughput experimentation, imaging technologies, multi‐omics analysis, and automated synthesis platforms. Realizing this vision will require close interdisciplinary collaboration among experts in data science, chemistry, engineering, biology, and clinical medicine. With such integrative efforts, the transition of nanomedicine from bench to bedside in cancer treatment is becoming increasingly attainable.

## Conflict of Interest

The authors declare no conflict of interest.
